# SGLT2 inhibitors in heart failure with tricuspid regurgitation: clinical interpretation and future directions

**DOI:** 10.1093/ehjcvp/pvag021

**Published:** 2026-04-11

**Authors:** Weikai Dong, Yebin Zhou, Junying Jiang

**Affiliations:** Cardiovascular Medical Center, Nanjing Drum Tower Hospital, the Affiliated Hospital of Nanjing University Medical School, Nanjing, Jiangsu Province 210008, China; School of Basic Medical Sciences & School of Public Health, Faculty of Medicine, Yangzhou University, Yangzhou 225009, China; Faculty of Medicine, Yangzhou University, Yangzhou 225009, China

To the Editor

We have read with great interest the recent publication by Loutati R *et al*., entitled ‘Heart Failure and Tricuspid Regurgitation: The Role of SGLT2 Inhibitors in Improving Outcomes’.^1^ This timely study used large real-world registration databases, which provided insights into populations with difficult clinical challenges and insufficient research, especially since past major randomized controlled trials excluded patients with severe valvular heart disease. The authors provided valuable real-world evidence from large registration databases, but several additional factors need to be noted to place the study findings in a broader context and make them more compelling.

Firstly, the study did not properly handle two factors that are relatively important for clinical heterogeneity, namely the timing of treatment and patient selection. The echocardiographic subgroup analysis of the EMPAG-HF trial has evidence that early use of empagliflozin can rapidly improve the left atrial and ventricular filling kinetics, which provides some ideas for explaining the mechanism of the reduced rehospitalization rate and mortality in high-risk acute heart failure patients.^2^

Secondly, little is known of the SGLT2i-arrhythmia connection. The lack of rhythm-related endpoints is unfortunate given the established relationship between tricuspid regurgitation (TR), right atrial dilation, and atrial fibrillation. Evidence from randomized controlled trials shows that SGLT2 inhibitors reduce atrial fibrillation in different cardiovascular populations.^3^ Therefore, SGLT2 inhibition may be associated with changes in rhythm disturbances. Including arrhythmic outcomes could help determine if part of the observed benefit is mediated via electrical remodelling rather than haemodynamic change.

Thirdly, uncertainty exists about whether results generalize to cardiovascular patients at large. SGLT2 inhibitors consistently reduce the risk of heart disease or stroke and the corresponding death in various patients, including high-risk non-diabetic populations and untreated diabetic patients.^4^ This obvious wide range of benefits indicates that the cardioprotective effect of SGLT2 inhibitors is not only effective in advanced heart failure patients but may also play a role through mechanisms related to the progression of general cardiovascular diseases.

Additionally, real-world eligibility and implementation gaps were not examined. Registration data show that many patients with heart failure in reality do not meet the eligibility for SGLT2 inhibitor trials, especially in trials such as SOLOIST-WHF, which reflects the gap between the trial population and routine clinical practice.^5^ Evaluating the consistency of eligibility and prescription patterns helps to determine whether the observed benefits are due to the drug's effect or factors in the healthcare system.

Lastly, the study did not involve SGLT2 inhibitors and transcatheter TR intervention. For the development of device therapy, it is important to be aware of the importance of combined or sequential treatment strategies for optimizing patient prognosis. Conclusion: This study promotes the application of SGLT2 inhibitors in heart failure patients with TR. Future studies need to increase the inclusion of drug comparative analysis, arrhythmia endpoints, wider populations, and real-world factors, and also need to integrate with intervention strategies to clarify its role in cardiovascular valve management. Our advice is geared towards improving an already excellent article and we look forward to more insightful articles from the authors.

## Author contributions

Weikai Dong: Writing—original draft, Writing—review & editing; Yebin Zhou and Junying Jiang: Supervision, Writing—review & editing.

AI statement: No generative artificial intelligence tools were utilized in the preparation of this article.

## Data Availability

No new data were generated or analysed in support of this research.
